# Highlights in the knowledge of brown spider toxins

**DOI:** 10.1186/s40409-017-0097-8

**Published:** 2017-02-08

**Authors:** Daniele Chaves-Moreira, Andrea Senff-Ribeiro, Ana Carolina Martins Wille, Luiza Helena Gremski, Olga Meiri Chaim, Silvio Sanches Veiga

**Affiliations:** 1 Department of Cell Biology, Federal University of Paraná (UFPR), Curitiba, PR Brazil; 2 Department of Structural and Molecular Biology, State University of Ponta Grossa (UEPG), Ponta Grossa, PR Brazil

**Keywords:** Brown spider, *Loxosceles*, Venom, Toxins, Loxoscelism, Phospholipase-D, Metalloprotease, Insecticidal peptides, Serineprotease, Hyaluronidase

## Abstract

Brown spiders are venomous arthropods that use their venom for predation and defense. In humans, bites of these animals provoke injuries including dermonecrosis with gravitational spread of lesions, hematological abnormalities and impaired renal function. The signs and symptoms observed following a brown spider bite are called loxoscelism. Brown spider venom is a complex mixture of toxins enriched in low molecular mass proteins (4–40 kDa). Characterization of the venom confirmed the presence of three highly expressed protein classes: phospholipases D, metalloproteases (astacins) and insecticidal peptides (knottins). Recently, toxins with low levels of expression have also been found in *Loxosceles* venom, such as serine proteases, protease inhibitors (serpins), hyaluronidases, allergen-like toxins and histamine-releasing factors. The toxin belonging to the phospholipase-D family (also known as the dermonecrotic toxin) is the most studied class of brown spider toxins. This class of toxins single-handedly can induce inflammatory response, dermonecrosis, hemolysis, thrombocytopenia and renal failure. The functional role of the hyaluronidase toxin as a spreading factor in loxoscelism has also been demonstrated. However, the biological characterization of other toxins remains unclear and the mechanism by which *Loxosceles* toxins exert their noxious effects is yet to be fully elucidated. The aim of this review is to provide an insight into brown spider venom toxins and toxicology, including a description of historical data already available in the literature. In this review article, the identification processes of novel *Loxosceles* toxins by molecular biology and proteomic approaches, their biological characterization and structural description based on x-ray crystallography and putative biotechnological uses are described along with the future perspectives in this field.

## Background

Since the brown spider, an arachnid of the genus *Loxosceles* (Araneae, Sicariidae), can be found worldwide, it has different common names depending on the region it is found, including brown recluse, violin spider and fiddleback spider [1–4]. The *Loxosceles* genus was described by Heineken and Lowe in 1832 [3, 5]. These spiders are brown in color with a characteristic dark violin-shaped mark on cephalothorax and have six equal sized eyes distributed in semi-circular fashion [6, 7]. The individuals present sexual dimorphism, the females usually have larger abdomens and can inject more venom when they bite [2]. Brown spiders are commonly found in workplaces with secluded, dry, sheltered areas such as underneath structures, logs, or in piles of rocks or leaves. The brown spider is also adapted to live indoors, they can be found in dark closets, inside shoes, or attics [6, 7]. Even though the genus *Loxosceles* comprises approximately 130 species and all of them are probably capable of producing clinically significant bites, the species responsible for envenomation in the United States are *Loxosceles reclusa*, *Loxosceles deserta* and *Loxosceles arizonica*. In Brazil, *Loxosceles intermedia*, *Loxosceles gaucho* and *Loxosceles laeta* are considered to be the most important spiders from the medical point of view [4, 8–11]. Spider envenomation is a serious public health threat in Brazil due to the number of cases recorded annually [12]. In 2015, 26,298 spider bites were recorded in Brazil, including 30 fatal cases [13]. Spiders of the *Loxosceles* genus are one of the four groups of spiders that produce venoms that can cause significant clinical manifestations in human or even fatalities following envenomation [14]. The condition that commonly appears after accidents involving *Loxosceles* spiders is known as loxoscelism and is characterized by several reactions. Although most bites are benign and local, systemic symptoms can emerge [6]. Local reactions include dark blue-violet colored necrotic wounds with gravitational spread, which eventually become indurated, and ultimately lead to scarring [2, 8]. In nearly half of the cases, cutaneous lesions are associated with non-specific systemic symptoms, including fever, fatigue, headache, vomiting, pruritus and rash [8, 11, 15]. Systemic loxoscelism is a less frequent complication (occurring in up to 13% of the cases) that usually affects children, and leads to manifestations such as renal failure and hematological disturbances, i.e., disseminated intravascular coagulation and intravascular hemolysis [7, 11, 16, 17]. The first clinical cases of loxoscelism were published in the literature describing both cutaneous and cutaneous-visceral reactions [18–20].

The treatment for loxoscelism includes mainly antivenom, corticosteroids and dapsone. However, there are no clinical trials to substantiate any method. In addition, it is difficult to evaluate the efficacy of the treatment because of the diverse forms of cutaneous lesions and often late diagnosis. While systemic corticosteroids are widely used in Brazil – either alone or associated with the antivenom – dapsone is frequently used in the USA, although there is no consensus on the efficacy of these treatments [21].

Indications for antivenom therapy depend mainly on the time of progression – the earlier the therapy is performed the greater the efficacy. This was corroborated by an experimental study that showed that necrotic injuries in rabbits were about 90% smaller compared with the control when the antivenom was administered up to 6 h, while the reduction in the lesion dropped to 30% when the antivenom was administered up to 48 h after the bite [22]. Health protocols in Brazil, Peru and Argentina advise the use of intravenous antivenom in cases of cutaneous or cutaneous-hemolytic forms of loxoscelism – when hemolysis is present the antivenom is indicated even 48 h after the bite [21].

However, antivenom therapy may lead to anaphylactic reactions. A clinical study showed that almost one third of the patients who received antivenom manifested some type of early anaphylactic reaction [23]. Experimental studies demonstrate some efforts in this direction by developing alternative means to elicit a protective immune response against the noxious effects of dermonecrotic toxins, such as using an immunogenic synthetic peptide or a neutralizing monoclonal antibody that protect rabbits mainly against dermonecrotic toxin activity [24, 25]. In this context, another study deepened this issue when it identified peptide epitopes of representative toxins in three species of *Loxosceles* describing new antigenic regions important to induce neutralizing antibodies. These synthetic peptides where used to develop an in vitro method to evaluate the neutralizing potency of horse hyperimmune sera (anti-*Loxosceles* sera) [26].

Epitopes of a recombinant dermonecrotic toxin from *L. intermedia* venom were also used to construct a chimeric protein called rCpLi. In this study, the authors demonstrate that horses immunized with three initial doses of crude venom followed by nine doses of rCpLi generate antibodies with the same reactivity as those produced following immunization exclusively with whole venom. They argue that the use of this new generation of antivenoms will reduce the suffering of horses and devastation of arachnid fauna [27].

Diagnosis of loxoscelism is difficult and usually presumptive. It is often made through evolution of the clinical picture and epidemiological information, since few patients bring the animal for its identification [23]. Recently, an experimental study developed a recombinant immunotracer based on a monoclonal antibody that reacts with *L. intermedia* venom components of 32–35 kDa and neutralizes the dermonecrotic activity of the venom. This antibody was re-engineered into a colorimetric bifunctional protein (antibody fragment fused to alkaline phosphatase) that proved to be efficient in two stated immunoassays. This immunotracer could become a valuable tool to develop immunoassays that may facilitate a rapid and reliable diagnostic of loxoscelism [28].﻿ As the cases of loxoscelism became noteworthy, *Loxosceles* spider venoms started to be investigated and biologically and biochemically characterized﻿﻿. This review is focused on different aspects of venom components, such as studies in toxinology employing 'omics' strategies and recombinant toxins. The following sections present a historical perspective of the accumulated knowledge regarding the brown spider venom.

## History of the brown spider venom toxinology

### Beginning of the venom study

**Fig. 1:**
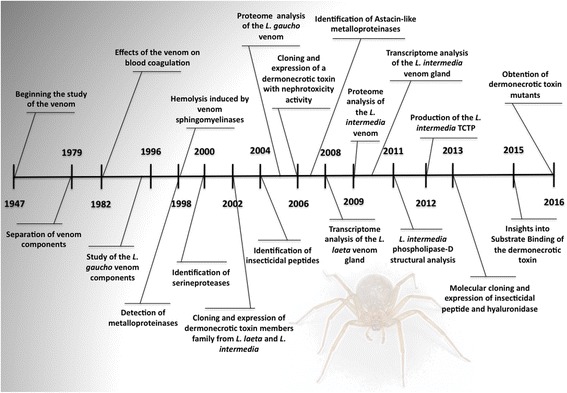
Major historical evolution on the knowledge on brown spider venom. Main publications in toxinology on *Loxosceles* spiders

**Fig. 2:**
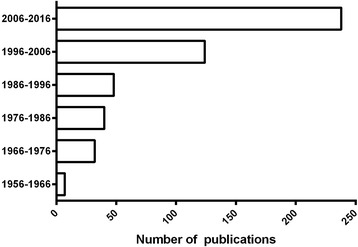
Number of scientific publications on *Loxosceles* during the last 60 years. Graphs were prepared using the number of articles retrieved in PubMed (http://www.ncbi.nlm.nih.gov/pubmed) using *‘Loxosceles’* in all fields as filter of search, in July 2016

*Loxosceles* spider venoms have been studied for over 60 years (Fig. 1). Different scientific research groups across the world started the process of venom extraction and characterization, motivated by the several reports of human loxoscelism cases. Earlier, due to technical limitations, the studies were based only on the in vitro and in vivo experimental observations. These observations yielded insight into the pathophysiology of cutaneous arachnoidism. The first experimental study of loxoscelism available in the literature was described by Macchiavello in 1947 [29]. That report described the stages of dermonecrosis in guinea pigs after spontaneous bite by *Loxosceles laeta*. The first studied venom of brown spider was extracted from *Loxosceles laeta* and, afterwards, from *Loxosceles reclusa* [29–32]. Since then, several studies on *Loxosceles* venoms and toxins were published and this subject attracted the attention of several scientists and research groups (Fig. 2).

### Separation of the venom components

During the end 1960’s and early 1970’s extraction of brown spider venom started along with isolation of individual components [33, 34]. According to the observations of Morgan in 1969 [34], the clear, highly viscous venom extracted from an adult female spider contained on average 50 μg of protein. Moreover, the venom extracted from eight males and eight females of *L. reclusa* spiders were determined by SDS-PAGE electrophoresis and were analyzed [35]. These *Loxosceles* venoms presented a similar protein profile and were enriched in low molecular mass protein molecules. Molecular mass analysis revealed three main groups of proteins with different molecular masses −30-40 kDa, 20–30 kDa and 2–10 kDa [35, 36]. The toxicity profiles of *Loxosceles* venoms were similar between female and male specimens, and between distinct species, such as *L. laeta*, *L. reclusa*, *L. intermedia*, *L. adelaida*, *L. similis* and *L. gaucho*. Partial purification of the venom toxins by sephadex gel filtration revealed three major fractions; fraction A, with hyaluronidase activity; fraction B, responsible for major dermonecrotic activity; and fraction C, devoid of dermonecrotic activity [33, 34, 37–40]. Furthermore, protease, esterase, and alkaline phosphatase activities were reported in *Loxosceles* venom [35–39, 41].

### Demonstration of the biological effects of the venom

The number of investigations, regarding the toxicity and pathophysiological effects of *Loxosceles* venom, increased together with the development of scientific techniques. The use of preparative gel electrophoresis and gel filtration provided tools for investigation of each protein fraction from brown spider venom [42–44]. Cation-exchange chromatography at pH 4.0 purified the toxin fraction responsible for lethality in mice, induction of necrosis in rabbits, calcium-dependent hemolysis of human erythrocytes, and a decrease in the calcium-induced coagulation time of human plasma [45]. Indeed, a fraction of the *L. reclusa* venom has also shown to produce hematological effects in albino mice [46, 47]. Similar effects were observed with *L. laeta* venom in rabbits. There were studies that demonstrated abnormalities in the blood coagulation process, including alterations in thromboplastin time, prothrombin time, platelet count and fibrinogen-fibrin degradation [48]. Moreover, a low molecular mass peptide fraction of *L. reclusa* venom was shown to contain lethal and neuroactive components to insects [49].

Despite the significance of studying protein fractions of brown spider venom, some recent and relevant studies focus on the mechanics of action of whole venom even though sometimes making a parallel with specific toxins. Systemic loxoscelism, for example, was the subject of two studies that focused on renal and cardiac toxicity [50, 51]. It was observed that *L. gaucho* venom caused early acute kidney injury in rats probably due to an impaired renal flow and systemic rhabdomyolysis. The authors also showed that renal damage is independent of a dermonecrotic injury or blood pressure changes [51]. Moreover, cardiotoxic effects of *L. intermedia* venom were studied in mice and results demonstrated that venom antigens were detected in the heart and that the venom induced an impairment in the heart function. The authors argue that these cardiotoxic effects could play a role in the symptoms of systemic loxoscelism, and that loxtox proteins are important to develop the heart dysfunction in envenomed mice [50].

Aiming to investigate the vascular disorders often associated with venom exposure, Nowatzki et al. [52, 53] analyzed the effects of *L. intermedia* venom on endothelial cells in culture in two different studies. They showed that the venom primarily induces specific changes to cellular adhesion followed by cell retraction, detachment and, finally, drives an apoptotic mechanism known as anoikis. These effects may lead to capillary vessel fragility and facilitate the observed hemorrhagic outcome [53]. Moreover, endothelial cell endocytosed the toxins of *L. intermedia* venom but, as no lysosomal damage was observed, the authors argue that deleterious effects on these cells are not caused by internalization of toxins [52]. Cultured keratinocytes exposed to *L. laeta* venom increased the expression/secretion of MMP2, MMP9 and MMP7, which was associated with cell death. These effects upon keratinocytes are likely to contribute to the pathology of cutaneous loxoscelism [54].

The release of inflammatory mediators after inoculation of *L. gaucho* venom in mice footpads was investigated and results showed a marked PGE_2_ release associated with an increase of interleukin-6 (IL-6), monocyte chemoattractant protein-1 (MCP-1) and keratinocyte chemoattractant (KC). Edema and leukocyte migration to the site of inoculation was also observed, thus suggesting that these mediators contribute to the inflammatory reaction induced by *L. gaucho* venom [55]. Platelets were also shown to have a role in inflammation, besides being also involved in local thrombotic disorders induced by *Loxosceles* venom. *L. gaucho* venom induced aggregation of platelets, activated adhesion to collagen and increased the expression of ligand-induced binding site 1 (LIBS1) and P-selectin, demonstrating the pivotal role of platelets in the development of dermonecrosis [56]. On the other hand, another study showed that the platelets have a role in minimizing the hemorrhagic phenomena and the inflammatory and wound-healing processes, since platelet depleted rabbits showed more severe reactions after *Loxosceles* venom application [57]. Despite all these studies demonstrating important mechanisms by which *Loxosceles* venom lead to the main injuries observed after envenomation, it is known that the venom is a mixture of several hundred biologically active compounds that act synergistically. Thus, the detailed mechanism of action of *Loxosceles* venoms remains unknown and is still object of study.

### Biochemical characterization of the venom components

Barbaro et al. [58], in 1992, used gel filtration to identify a 35-kDa fraction of *L. gaucho* venom. This fraction was found to have dermonecrotic, immunogenic and life-threatening activities; it was also the first antigen to be detected by antibodies during the course of immunization. This 35-kDa fraction purified from *L. intermedia* venom was found to be able to be incorporated into human erythrocytes membranes and render them susceptible to the alternative pathway of complement. A functional analysis of this venom fraction indicated the presence of sphingomyelinase activity and that it was capable of inducing all the in vivo effects seen with whole spider venom, including C-dependent hemolysis and dermonecrosis [59].

Protease activities were also found in brown spider venoms, with distinct molecular mass profiles and substrate preferences [60, 61]. Based on the enzymatic features, they were classified as metalloproteases and serinoproteases. Two brown spider metalloproteases were identified, namely loxolysin A (20 kDa), with activity on fibronectin and fibrinogen, and loxolysin B (30 kDa), with gelatinolytic activities [60]. Regarding the presence of metalloproteases in *Loxosceles* venom, two proteases were also found in *L. rufescens* venom, a 23-kDa fibrogenolytic protease and a 27-kDa gelatinolytic protease. Their activities were inhibited by 1,10-phenantroline, confirming the metalloprotease characteristic of the protease [62, 63]. The degradation of fibrinogen was reported to occur due to different *Loxosceles* venoms; again, inhibition of degradation by 1,10-phenantroline was also reported [64, 65].

Serineproteases were detected in *L. intermedia* venom by zymographic assays showing two gelatinolytic signals with high molecular masses (85 kDa and 95 kDa) [61]. The biochemical nature of these proteases was characterized by total inhibition of gelatin hydrolysis using distinct serineprotease inhibitors such as aprotinin, benzamidine, leupeptin, PMSF, and soybean-trypsin inhibitor [61].

Later on, the first description of peptides from the inhibitor cystine knot family (ICK) in *Loxosceles* venoms was published by de Castro et al. [66]. These small peptides isolated from the venom of *L. intermedia* demonstrated insecticidal activities, and were named LiTx1, LiTx2, and LiTx3. These components are polypeptides with molecular masses ranging from 5.6 to 7.9 kDa, presenting insecticidal activities against highly destructive pests such as *Spodoptera frugiperda* and *Spodoptera cosmioides*. Further analysis of the sequences pointed to the presence of possible post-translational modification regions in the sequences of LiTx1-3, such as N-myristoylation, amidation, and casein kinase II phosphorylation sites. Based on the sequences of these toxins, the authors proposed that LiTx-3 may act on NaV (voltage-gated sodium) channels and that LiTx-2 and 3 may act on NaV or CaV (voltage-sensitive calcium) channels [66].

### Omics and recombinant venom components

**Fig. 3:**
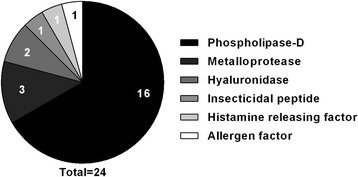
*Loxosceles* recombinant toxins. Graph shows the percentage of recombinant *Loxosceles* toxins described in the literature classified by class of toxins. In each type of toxin, the number of recombinant isoforms is available

Molecular biology techniques were essential for understanding the toxicology of *Loxosceles* venoms. The amount of venom (volume and protein) that can be extracted from each spider is small, hampering the process of isolation of single native toxins. The first toxin to be cloned and studied in the recombinant form was a sphingomyelinase-D from *L. laeta* venom in 2002 by Fernandes-Pedrosa et al. [67]. In the same year, Kalapothakis et al. [68] cloned and expressed a functional sphingomyelinase-D from *L. intermedia* spider venom and demonstrated its immunological properties. A characterization of a phospholipase D from *L. gaucho* was also reported [69]. Nowadays, there are 24 reports of recombinant toxins from *Loxosceles* in the literature (Fig. 3).

The *L. laeta* venom gland transcriptome analysis revealed that 16.4% of the total toxin-encoding ESTs belong to sphingomyelinases-D [70]. Recently it was found that 15% of the whole *L. similis* venom gland transcriptome corresponds to phospholipase-D transcripts [71]. Moreover, the *L. intermedia* transcriptome analysis revealed more than 20.2% of all toxin-encoding ESTs from *L. intermedia* venom gland correspond to phospholipases D and represents a significant proportion of the toxins present in the brown spider venom [72]. Corroborating these findings, two-dimensional gel electrophoresis demonstrated at least 25 spots immunologically related to phospholipases D toxins in *L. intermedia* crude venom [73]. Indeed, at least 11 phospholipase-D isoforms were identified in the venom proteome of *L. gaucho*, corroborating the presence of several different dermonecrotic toxins in the Brown spider venom [74].

Using RNA sequencing, 23 complete sequences of phospholipase-D proteins (PLD) were found in *L. similis* venom gland and classified as loxtox proteins [71, 75]. Seven different isoforms of phospholipase-D were generated as recombinant proteins, namely LiRecDT (*Loxosceles intermedia* recombinant dermonecrotic toxin) and these enzymes have also been classified as members of the LoxTox family [75–80]. Several other isoforms have also been identified in the venoms of *Loxosceles reclusa, Loxosceles laeta, Loxosceles arizonica, Loxosceles similis, Loxosceles boneti, and Loxosceles deserta* [81–89]. Studies comparing recombinant isoforms with distinct capacities for degrading substrates have demonstrated differences in the intensity of the effects of these proteins [90].

Most enzyme isoforms from the *Loxosceles* genus have been heterologously produced in prokaryotic systems using *E. coli,* and large amounts of the soluble and enzymatically active forms of these proteins are easily obtained. The knowledge of PLD sequences allowed the development of promising tools, such as a recombinant chimeric protein immunogen expressing epitopes of a dermonecrotic toxin from *L. intermedia* venom, which was atoxic and capable of inducing dermonecrotic and hemorrhagic protection [91]. Brown spider phospholipases D catalyzes the hydrolysis of phospholipids, such as sphingomyelin (SM), at a terminal phosphodiester bond to release choline and produce ceramide 1-phosphate (C1P) [73, 90, 92]. The catalysis mediated by phospholipases D in the presence of Mg^+2^-cofactor leads to hydrolysis of lysophosphatydilcholine (LPC) and release of lysophosphatidic acid (LPA) [81, 92, 93]. It seems that the production of these bioactive metabolites can promote upregulation of proinflammatory molecules and exert deleterious effects after exposure to brown spider phospholipases D [90, 92, 94–99].

Alternatively, some authors stand up for that phospholipase-D toxins (testing recombinant toxins and whole venoms) exclusively catalyze transphosphatidylation rather than hydrolysis, forming cyclic phosphate products from both major substrates – SM and LPC [100]. It was also shown that a sphingomyelinase-D from *Loxosceles arizonica* (Laz-SMase D) is a potent insecticidal toxin [101].

The first metalloprotease, cloned and expressed from the cDNA library, was extracted from *Loxosceles intermedia* venom gland, and was characterized as an astacin-like protease. This astacin metalloprotease presented a catalytic domain of 18 amino acids – HEXXHXXGXXHEXXRXDR – and a conserved methionine involved in a sequence turn, met-turn, and zinc-dependent activity (MXY) [102]. The recombinant *Loxosceles intermedia* astacin-like protease (LALP) promoted endothelial cell cultures de-adhesion, in vitro degradation of fibronectin, fibrinogen, and gelatin [63]. Astacin proteases comprises a family of toxins in *L. intermedia* venom, two other isoforms, named LALP2 and LALP3 were also described [103]. Besides, astacins were identified in *L. laeta* (LALP4) and *L. gaucho* (LALP5) venoms, suggesting the existence of an interspecies toxin family and revealing the importance of these metalloproteases as components of *Loxosceles* venom [104].

Interestingly, when transcriptome complete analysis of *L. intermedia* and *L. laeta* venom glands were performed these studies revealed that astacin metalloproteases are included among the high expressed toxins [70, 72]. In *L. intermedia* venom gland, astacin transcripts comprise more than 22% of the toxin-encoding transcripts and represent 8% of the total transcripts in *L. laeta* venom gland [70, 72]. *Loxosceles* proteases (metalloproteases and serineproteases) account for 23.1% of the total toxin-encoding transcripts in *L. intermedia* venom gland, second only to the insecticidal peptide sequences that comprise the majority of expressed toxins. In addition, the analysis of proteases in the *L. intermedia*, *L. laeta*, and *L. gaucho* venoms using two dimensional western blotting and zymogram, demonstrated a great content of active proteases among the three analyzed venoms, corroborating the high mRNA expression reported on the transcriptome analysis [104].

Regarding the ICK peptides in *Loxosceles* venom, transcriptome analysis of *L. intermedia* venom gland found that ICK peptides comprise 55.6% of toxin-encoding messengers [72]. Previously described ICK peptides (LiTx1-3) were found and a novel ICK peptide from *L. intermedia*, LiTx-4, was identified, and later described by the authors. The most abundant toxin transcripts found were transcripts similar to LiTx-3 (32%), LiTx-2 (11.4%), LiTx-1 (6.2%), and LiTx-4 (3.7%) [72].

In fact, it was reported that the cloning and production of a recombinant peptide from *L. intermedia* venom had a great similarity with the ICK family of peptides, especially LiTx-3 [105]. The recombinant peptide, named U2-sicaritoxin-Li1b (U2- SCRTX-Li1b), was used as a tool that enabled the demonstration of an antigenic cross-reactivity of antisera raised against crude venom of *L. intermedia*, *L. gaucho,* and *L. laeta* with U2-SCRTX-Li1b. This cross-reactivity corroborates the presence of ICK-like toxin members in these *Loxosceles* venoms, thus strengthening the idea that this toxin family is widespread throughout the genus [105, 106].

### Structural analysis of *Loxosceles* toxins

The first structural study on *Loxosceles* toxins was performed by Zela et al. in 2004 [107], in which the crystallization and preliminary crystallographic analysis of a sphingomyelinase-D from *L. laeta* spider venom were performed. Crystal structure of LiRecDT1 from *L. intermedia* was published by de Giuseppe et al. [108], indicating that this toxin contained an additional disulfide bond in the toxin structure catalytic loop compared with the previously described phospholipase-D from *L. laeta* [109, 110]. The phospholipase-D from *L. gaucho* was also crystallized by Ullah et al. [111] in 2014 and the structure was shown to be very similar to the phospholipase-D from *L. intermedia* [112]*.*

The structural details of the molecules reflect the distinct enzymatic behaviors of the venom from different species. Phospholipase-D with different structures could have different substrate affinities or enzymatic activities; therefore, these differences could explain the clinical symptoms or severity observed at the local bite site or the systemic effects during envenomation by different species of the *Loxosceles* genus. In addition, structural analysis of the catalytic site provided important insights into the enzymatic activities of each isoform [108, 110, 112].

Comparisons of the amino acid sequences of spider venom PLDs indicate that these proteins contain either 284 or 285 amino acids and display a significant degree of homology, mainly with regard to the catalytic important residues [85]. The single polypeptide chain folds to form a distorted TIM-barrel, which is lined with eight parallel β-strands internally linked by short flexible loops to eight α-helices that form the outer surface of the barrel [110]. The catalytic loop is stabilized by a disulfide bridge (Cys51 and Cys57) in the *L. laeta* and with a second disulfide bridge (Cys53 and Cys201) in the *L. intermedia*, which links the catalytic loop to the flexible loop to significantly reduce the flexibility of the latter loop [108–110]. The catalytic site, Mg^2+^ binding site, and the substrate binding site are located in a shallow depression that contain His12, Glu32, Asp34, Asp91, His47, Lys93, Tyr228, and Trp230, which are very conserved in *Loxosceles* PLD isoforms [108, 110]. The importance of theses residues was confirmed by site-directed mutagenesis and the X-ray structural studies indicating involvement of the two histidines (His12 and His47) in close proximity to the magnesium coordination (Glu32, Asp34, and Asp91) that promote the acid-base catalytic mechanism. Furthermore, the residues Lys93, Tyr228, and Trp230 were shown to be important for recognition and stabilization of the substrate (phospholipid) during the catalytic process [113, 114].

Several mutants of PLDs were studied recently bringing light in the understanding of the catalytic and recognition sites [114, 115]. However, the variety of molecular mechanisms triggered by *Loxosceles* phospholipase-D toxins and their lipid metabolites should be further investigated as a complex event dependent on the types of cells involved, the abundance, and availability of the lipid substrate, and intracellular and extracellular signaling cascades [97, 116]. For now, it is demonstrated that phospholipases D from different *Loxosceles* species have the ability to reproduce many effects of the cutaneous and cutaneous-visceral loxoscelism. They are described as being responsible for several biological properties ascribed to the whole venom, including dermonecrosis, massive inflammatory response with neutrophil infiltration, complement activation, platelet aggregation, immunogenicity, edema, increased vessel permeability, hemolysis, renal failure, toxicity for several cultured cell types, and animal lethality [65, 76–81, 84, 90, 92–95, 114, 117–120].

Recently, we have observed that all this deleterious events can be prevented using specific phospholipases inhibitors that can decrease the brown spider recombinant phospholipase-D activity [121]. This strengthen the idea of the importance of designing and optimizing a specific drug to treat the serious clinical symptoms caused by the brown spider bite, a public health problem in several parts of the world and until now without specific treatment.

### Production of novel and less expressed components in recombinant form

Serineproteases, hyaluronidases, venom allergens, a histamine releasing factor also known as translationally controlled tumor protein (TCTP), enzymatic inhibitors (serpins), and C-type lectins were identified in transcriptome studies of *Loxosceles* venom glands [70, 72]. The cDNA libraries enabled an overview of the *Loxosceles* venom and allowed the description of new molecules of biotechnological interest.

Since then, several components, i.e., TCTP and hyaluronidases were further explored and produced as recombinant molecules [122, 123]. New isoforms of the previously described and studied toxins served as tools that strengthened the knowledge concerning venom actions and loxoscelism [76, 78–80, 102, 104, 124].

The identification of hyaluronidase activity in *Loxosceles* venoms comes from a study of *L. reclusa* venom, which demonstrated hyaluronidase activity upon hyaluronic acid (HA) and condroitin-sulphate (CS) types A, B, and C [39]. The medically important venoms from five *Loxosceles* species in the US (*L. deserta*, *L. gaucho*, *L. intermedia*, *L. laeta,* and *L. reclusa*) contain a 44-kDa hyaluronidase, which is able to degrade HA detected by zymogram assays [65]. All these identifications of *Loxosceles* hyaluronidases suggest the biological conservation and significance of these enzymes [65]. Two hyaluronidase molecules of 41 and 43 kDa were characterized as pH-dependent endo-β-N-acetyl-d-hexosaminidases hydrolases in *L. intermedia* venom [124]. These enzymes were able to degrade HA and CS in vitro and HA in rabbit skin [124].

Corroborating the identification of hyaluronidase activity, a proteomic study also described the presence of hyaluronidases in *Loxosceles* venoms [125]. *Loxosceles* hyaluronidase shows high activity, requiring few micrograms of venom to demonstrate its activity [40, 65, 124]. The transcriptome analysis of *L. laeta* and *L. intermedia* venom glands showed that this class of toxin is minimally expressed representing only 0.13% of the total expressed sequences of *L. laeta* venom gland [70, 72]. A brown spider recombinant hyaluronidase from *L. intermedia* venom presenting a molecular mass of 46 kDa was obtained and characterized [122]. The active enzyme, after in vitro refolding, was able to degrade HA and CS. These results corroborate previous data concerning a native hyaluronidase that degrades both glycosaminoglycans demonstrating that the recombinant hyaluronidase can also be considered as chondroitinase [122]. The biological characterization of the recombinant hyaluronidase showed an increase in erythema, ecchymosis, and dermonecrotic effects induced by the recombinant dermonecrotic toxin (LiRecDT1) in rabbit skin [122]. Furthermore, a new *Loxosceles intermedia* hyaluronidase isoform (42 kDa) was successfully expressed and secreted by insect cells (SF-9) by baculovirus technology. This novel toxin presented activity against HA and its characterization is in process (Chaves-Moreira: personal communication).

The *L. intermedia* venom gland transcriptome analysis described the sequence of a protein identified as possible histamine releasing factor (HRF/TCTP) expressed at relatively low level in the venom, i.e., only 0.4% of the toxin-encoding transcripts [72]. The functional characterization of the recombinant protein, called LiTCTP, revealed that this toxin leads to edema and enhanced vascular permeability [123]. The cutaneous symptoms of envenomation with *Loxosceles* venom include erythema, itching and pain. In some cases, *Loxosceles* spider bites can cause hypersensitivity or even allergic reactions. These responses could be associated with histaminergic events, such as an increase in vascular permeability and vasodilatation. LiTCTP could be associated with these deleterious venom activities, as this protein was identified in *L. intermedia* venom. Another *Loxosceles* TCTP has been described in the venom gland of *Loxosceles laeta* using transcriptome analysis [70].

Sequences with significant similarity with allergen-like toxins from other venoms were found on the transcriptome studies of *L. laeta* and *L. intermedia* venom glands [70, 72]. These sequences described in *L. intermedia* transcriptome encode for venom allergens that are cysteine-rich molecules and show significant similarity to allergens from another spider genus (*Lycosa sigoriensis*), scorpions and mite allergens [72]. The amino acid sequence of a putative allergen from *L. laeta* venom is similar to venom allergen III and includes the presence of conserved cysteine residues [70]. In fact, allergic reactions following *Loxosceles* bites have been described in a few cases, as reviewed by Gremski et al. in 2014 [10]. A fine macular or papular eruption appears over the entire body in approximately 25% of the published cases of loxoscelism. In addition, cases of acute generalized exanthematous pustulosis (AGEP) after accidents with *L. reclusa* and *L. rufescens* have been reported [126, 127]. A recombinant allergen factor from *L. intermedia* venom was already cloned with a calculated molecular mass of 46 kDa and five disulfide bonds (Chaves-Moreira: personal communication). The expression of this recombinant protein will help to investigate the underlying mechanisms involved in the allergic responses observed in loxoscelism cases and might be used to biomedical purposes in this field.

## Conclusion

*Loxosceles* toxins are continuously being studied by researchers worldwide (Figs. 1 and 2). In recent years, a great amount of new toxins were identified in *Loxosceles* venom through combination of data from molecular biology techniques, proteomic studies, and characterization of recombinant toxins. Indeed, the identification, the biochemical and biological characterization and the structural studies of *Loxosceles* toxins improved the knowledge on venom composition and the involvement of these toxins in loxoscelism. However, there are many molecules (especially, those with low level of expression) that remain unidentified, without biological characterization and/or unknown mechanisms of action. Most of these unidentified molecules presented difficulties and solubility problems when prokaryotic expression systems were applied. Eukaryotic expression systems are proposed to ensure extraction of these toxins. Promising initial results were achieved with baculovirus and insect cells technology as well as with plant heterologous models for protein expression, as these models promoted extraction of soluble, pure and active forms of new toxins.

Therefore, further studies focusing on the recombinant production of novel toxins or the production of larger amounts of known toxins are imperative for characterization of their different components. *Loxosceles* toxicology can explore the putative biotechnological applications of toxins. The designing of inhibitor molecules for different toxins could be used as tools to elucidate the mechanisms of action and to elaborate protocols of basic and clinical research. It is of great interest to find inhibitors with the ability to stop or even delay the process of development and progression of loxoscelism as there is still no specific treatment available for the brown spider bite.

## Abbreviations

AGEP: Acute generalized exanthematous pustulosis
C1P: Ceramide 1-phosphate
CS: Condroitin-sulphate
HA: Hyaluronic acid
HRF: Histamine releasing factor
ICK: Inhibitor Cystine Knot family
IL-6: Interleukin-6
KC: Keratinocyte chemoattractant
LALP: Loxosceles intermedia astacin-like protease
LIBS1: Ligand-induced binding site 1
LPA: Release of lysophosphatidic acid
LPC: Lysophosphatydilcholine
MCP-1: Monocyte chemoattractant protein-1
PLD: Phospholipase-D
SM: Sphingomyelin
TCTP: Translationally controlled tumor protein

## References

[1] Vetter RS, Hinkle NC, Ames LM (2009). Distribution of the brown recluse spider (Araneae: Sicariidae) in Georgia with comparison to poison center reports of envenomations. J Med Entomol.

[2] da Silva PH, da Silveira RB, Appel MH, Mangili OC, Gremski W, Veiga SS (2004). Brown spiders and loxoscelism. Toxicon.

[3] Lucas SM (2015). The history of venomous spider identification, venom extraction methods and antivenom production: a long journey at the Butantan Institute, Sao Paulo, Brazil. J Venom Anim Toxins Incl Trop Dis.

[4] Cordeiro FA, Amorim FG, Anjolette FA, Arantes EC (2015). Arachnids of medical importance in Brazil: main active compounds present in scorpion and spider venoms and tick saliva. J Venom Anim Toxins Incl Trop Dis.

[5] Platinick NI. The world spider catalog. Bull Am Mus Nat Hist. 2013;14(5).

[6] Futrell JM (1992). Loxoscelism. Am J Med Sci.

[7] Hogan CJ, Barbaro KC, Winkel K (2004). Loxoscelism: old obstacles, new directions. Ann Emerg Med.

[8] Chaim OM, Trevisan-Silva D, Chaves-Moreira D, Wille AC, Ferrer VP, Matsubara FH, Mangili OC, da Silveira RB, Gremski LH, Gremski W (2011). Brown spider (*Loxosceles* genus) venom toxins: tools for biological purposes. Toxins (Basel).

[9] Chaves-Moreira D, Trevisan-Silva D, Gremski LH, Veiga SS. Brown spider venom: the identification and biotechnological potential of venom toxins. In: Venom genomics and proteomics. Edited by Gopalakrishnakone P. Dordrecht: Springer Netherlands; 2014. p. 1–22.

[10] Gremski LH, Trevisan-Silva D, Ferrer VP, Matsubara FH, Meissner GO, Wille AC, Vuitika L, Dias-Lopes C, Ullah A, de Moraes FR (2014). Recent advances in the understanding of brown spider venoms: From the biology of spiders to the molecular mechanisms of toxins. Toxicon.

[11] Isbister GK, Fan HW (2011). Spider bite. Lancet.

[12] Chippaux JP (2015). Epidemiology of envenomations by terrestrial venomous animals in Brazil based on case reporting: from obvious facts to contingencies. J Venom Anim Toxins Incl Trop Dis.

[13] SINAN, SUS. Casos de acidentes por aranhas no Brasil. In *Ministério da Saúde* (Notificação SdIdAd ed., 2015 edition. Brasilia; 2015.

[14] Isbister GK, Vetter RS (2005). Loxoscelism and necrotic arachnidism: more myths and minor corrections. Ann Emerg Med.

[15] Dandoy C, Grimley M (2014). Secondary hemophagocytic lymphohistiocytosis (HLH) from a presumed brown recluse spider bite. J Clin Immunol.

[16] Swanson DL, Vetter RS (2006). Loxoscelism. Clin Dermatol.

[17] Vetter RS, Isbister GK (2008). Medical aspects of spider bites. Annu Rev Entomol.

[18] Meneghello J, Emparanza E (1952). Cutaneo-visceral complications of Loxosceles laeta bite and cortisone; report of a case. Rev Chil Pediatr.

[19] Donckaster R, Cohen H (1960). A case of *Loxosceles* poisoning difficult to diagnosis. Bol Chil Parasitol.

[20] Schenone H, Semprevivo L, Schirmer E (1959). Two cases of cutaneo-visceral loxoscelism. Bol Chil Parasitol.

[21] Malaque CMS, Chaim OM, Entres M, Barbaro KC, Gopalakrishnakone P, Corzo G, de Lima MH, Diego-García E (2016). **Loxosceles and loxoscelism: biology, venom, envenomation, and treatment**. Spider venoms.

[22] Pauli I, Minozzo JC, da Silva PH, Chaim OM, Veiga SS (2009). Analysis of therapeutic benefits of antivenin at different time intervals after experimental envenomation in rabbits by venom of the brown spider *(Loxosceles intermedia*). Toxicon.

[23] Malaque CM, Santoro ML, Cardoso JL, Conde MR, Novaes CT, Risk JY, Franca FO, de Medeiros CR, Fan HW (2011). Clinical picture and laboratorial evaluation in human loxoscelism. Toxicon.

[24] Dias-Lopes C, Guimaraes G, Felicori L, Fernandes P, Emery L, Kalapothakis E, Nguyen C, Molina F, Granier C, Chavez-Olortegui C (2010). A protective immune response against lethal, dermonecrotic and hemorrhagic effects of *Loxosceles intermedia* venom elicited by a 27-residue peptide. Toxicon.

[25] Dias-Lopes C, Felicori L, Rubrecht L, Cobo S, Molina L, Nguyen C, Galea P, Granier C, Molina F, Chavez-Olortegui C (2014). Generation and molecular characterization of a monoclonal antibody reactive with conserved epitope in sphingomyelinases D from *Loxosceles* spider venoms. Vaccine.

[26] Ramada JS, Becker-Finco A, Minozzo JC, Felicori LF, Machado de Avila RA, Molina F, Nguyen C, de Moura J, Chavez-Olortegui C, Alvarenga LM (2013). Synthetic peptides for in vitro evaluation of the neutralizing potency of *Loxosceles* antivenoms. Toxicon.

[27] Figueiredo LF, Dias-Lopes C, Alvarenga LM, Mendes TM, Machado-de-Avila RA, McCormack J, Minozzo JC, Kalapothakis E, Chavez-Olortegui C (2014). Innovative immunization protocols using chimeric recombinant protein for the production of polyspecific loxoscelic antivenom in horses. Toxicon.

[28] Jiacomini I, Silva SK, Aubrey N, Muzard J, Chavez-Olortegui C, De Moura J, Billiald P, Alvarenga LM (2016). Immunodetection of the “brown” spider (*Loxosceles intermedia*) dermonecrotoxin with an scFv-alkaline phosphatase fusion protein. Immunol Lett.

[29] Macchiavello A (1947). Cutaneous arachnoidism experimentally produced with the glandular poison of *Loxosceles laeta*. PR J Public Health Trop Med.

[30] Vellard J (1956). Action in vitro of the venom of the South American spider *Loxosceles laeta*. C R Hebd Seances Acad Sci.

[31] Vellard J (1956). Venom of the spider *Loxosceles laeta* (Nic). C R Hebd Seances Acad Sci.

[32] Denny WF, Dillaha CJ, Morgan PN (1964). Hemotoxic effect of *Loxosceles reclusus* venom: in vivo and in vitro studies. J Lab Clin Med.

[33] Smith CW, Micks DW (1968). A comparative study of the venom and other components of three species of *Loxosceles*. Am J Trop Med Hyg.

[34] Morgan PN (1969). Preliminary studies on venom from the brown recluse spider *Loxosceles reclusa*. Toxicon.

[35] Norment BR, Jarratt JH (1976). Electrophoresis and electrofocusing of venom of L*oxosceles reclusa* Gertsch & Mulaik (Araneida: Scytodidae). J Med Entomol.

[36] Geren CR, Chan TK, Howell DE, Odell GV (1976). Isolation and characterization of toxins from brown recluse spider venom (*Loxosceles reclusa*). Arch Biochem Biophys.

[37] Suarez G, Biggemann U, Schenone H (1971). Biochemical study of the venom of *Loxosceles laeta* and of its mechanism of action. Bol Chil Parasitol.

[38] Suarez G, Schenone H, Socias T (1971). *Loxosceles laeta* venom – partial purification. Toxicon.

[39] Wright RP, Elgert KD, Campbell BJ, Barrett JT (1973). Hyaluronidase and esterase activities of the venom of the poisonous brown recluse spider. Arch Biochem Biophys.

[40] Bordon KC, Wiezel GA, Amorim FG, Arantes EC (2015). Arthropod venom hyaluronidases: biochemical properties and potential applications in medicine and biotechnology. J Venom Anim Toxins Incl Trop Dis.

[41] Heitz JR, Norment BR (1974). Characteristics of an alkaline phosphatase activity in brown recluse venom. Toxicon.

[42] Norment BR, Jong YS, Heitz JR (1979). Separation and characterization of venom components in *Loxosceles reclusa* – III. Hydrolytic enzyme activity. Toxicon.

[43] Jong YS, Norment BR, Heitz JR (1979). Separation and characterization of venom components in the brown recluse spider (*Loxosceles reclusa*) – I. Preparative disc electrophoresis. Toxicon.

[44] Jong YS, Norment BR, Heitz JR (1979). Separation and characterization of venom components in *Loxosceles reclusa* – II. Protease enzyme activity. Toxicon.

[45] Babcock JL, Civello DJ, Geren CR (1981). Purification and characterization of a toxin from brown recluse spider (*Loxosceles reclusa*) venom gland extracts. Toxicon.

[46] Chu JY, Rush CT, O'Connor DM (1978). Hemolytic anemia following brown spider (*Loxosceles reclusa*) bite. Clin Toxicol.

[47] Moran O, Zavaleta A, Castro dela Mata R (1981). Hematological effects of *Loxosceles laeta* venom in albino mice. Bol Chil Parasitol.

[48] Bascur L, Yevenes I, Barja P (1982). Effects of *Loxosceles laeta* spider venom on blood coagulation. Toxicon.

[49] Foil LD, Frazier JL, Norment BR (1979). Partial characterization of lethal and neuroactive components of the brown recluse spider (*Loxosceles reclusa*) venom. Toxicon.

[50] Dias-Lopes C, Felicori L, Guimaraes G, Gomes ER, Roman-Campos D, Duarte H, Damasceno D, Martins M, Kalapothakis E, Almeida AP (2010). Cardiotoxic effects of *Loxosceles intermedia* spider venom and the recombinant venom toxin rLiD1. Toxicon.

[51] Lucato RV, Abdulkader RC, Barbaro KC, Mendes GE, Castro I, Baptista MA, Cury PM, Malheiros DM, Schor N, Yu L, Burdmann EA (2011). *Loxosceles gaucho* venom-induced acute kidney injury – in vivo and in vitro studies. PLoS Negl Trop Dis.

[52] Nowatzki J, de Sene RV, Paludo KS, Veiga SS, Oliver C, Jamur MC, Nader HB, Trindade ES, Franco CR (2010). Brown spider venom toxins interact with cell surface and are endocytosed by rabbit endothelial cells. Toxicon.

[53] Nowatzki J, Sene RV, Paludo KS, Rizzo LE, Souza-Fonseca-Guimaraes F, Veiga SS, Nader HB, Franco CR, Trindade ES (2012). Brown spider (*Loxosceles intermedia*) venom triggers endothelial cells death by anoikis. Toxicon.

[54] Correa MA, Okamoto CK, Goncalves-de-Andrade RM, van den Berg CW, Tambourgi DV (2016). Sphingomyelinase D from *Loxosceles laeta* venom induces the expression of MMP7 in human keratinocytes: contribution to dermonecrosis. PLoS One.

[55] Barbaro KC, Lira MS, Araujo CA, Pareja-Santos A, Tavora BC, Prezotto-Neto JP, Kimura LF, Lima C, Lopes-Ferreira M, Santoro ML (2010). Inflammatory mediators generated at the site of inoculation of *Loxosceles gaucho* spider venom. Toxicon.

[56] Tavares FL, Peichoto ME, Rangel Dde M, Barbaro KC, Cirillo MC, Santoro ML, Sano-Martins IS (2011). *Loxosceles gaucho* spider venom and its sphingomyelinase fraction trigger the main functions of human and rabbit platelets. Hum Exp Toxicol.

[57] Tavares FL, Peichoto ME, Marcelino JR, Barbaro KC, Cirillo MC, Santoro ML, Sano-Martins IS (2016). Platelet participation in the pathogenesis of dermonecrosis induced by *Loxosceles gaucho* venom. Hum Exp Toxicol.

[58] Barbaro KC, Cardoso JL, Eickstedt VR, Mota I (1992). Dermonecrotic and lethal components of *Loxosceles gaucho* spider venom. Toxicon.

[59] Tambourgi DV, Magnoli FC, van den Berg CW, Morgan BP, de Araujo PS, Alves EW, Da Silva WD (1998). Sphingomyelinases in the venom of the spider *Loxosceles intermedia* are responsible for both dermonecrosis and complement-dependent hemolysis. Biochem Biophys Res Commun.

[60] Feitosa L, Gremski W, Veiga SS, Elias MC, Graner E, Mangili OC, Brentani RR (1998). Detection and characterization of metalloproteinases with gelatinolytic, fibronectinolytic and fibrinogenolytic activities in brown spider (*Loxosceles intermedia*) venom. Toxicon.

[61] Veiga SS, da Silveira RB, Dreyfus JL, Haoach J, Pereira AM, Mangili OC, Gremski W (2000). Identification of high molecular weight serine-proteases in *Loxosceles intermedia* (brown spider) venom. Toxicon.

[62] Young AR, Pincus SJ (2001). Comparison of enzymatic activity from three species of necrotising arachnids in Australia: *Loxosceles rufescens*, *Badumna insignis* and *Lampona cylindrata*. Toxicon.

[63] da Silveira RB, dos Santos Filho JF, Mangili OC, Veiga SS, Gremski W, Nader HB, von Dietrich CP (2002). Identification of proteases in the extract of venom glands from brown spiders. Toxicon.

[64] Zanetti VC, da Silveira RB, Dreyfuss JL, Haoach J, Mangili OC, Veiga SS, Gremski W (2002). Morphological and biochemical evidence of blood vessel damage and fibrinogenolysis triggered by brown spider venom. Blood Coagul Fibrinolysis.

[65] Barbaro KC, Knysak I, Martins R, Hogan C, Winkel K (2005). Enzymatic characterization, antigenic cross-reactivity and neutralization of dermonecrotic activity of five *Loxosceles* spider venoms of medical importance in the Americas. Toxicon.

[66] de Castro CS, Silvestre FG, Araujo SC, de MY G, Mangili OC, Cruz I, Chavez-Olortegui C, Kalapothakis E (2004). Identification and molecular cloning of insecticidal toxins from the venom of the brown spider *Loxosceles intermedia*. Toxicon.

[67] Fernandes-Pedrosa F, Junqueira de AzevedoIde L, Goncalves-de-Andrade RM, van den Berg CW, Ramos CR, Ho PL, Tambourgi DV (2002). Molecular cloning and expression of a functional dermonecrotic and haemolytic factor from *Loxosceles laeta* venom. Biochem Biophys Res Commun.

[68] Kalapothakis E, Araujo SC, de Castro CS, Mendes TM, Gomez MV, Mangili OC, Gubert IC, Chavez-Olortegui C (2002). Molecular cloning, expression and immunological properties of LiD1, a protein from the dermonecrotic family of *Loxosceles intermedia* spider venom. Toxicon.

[69] Magalhaes GS, Caporrino MC, Della-Casa MS, Kimura LF, Prezotto-Neto JP, Fukuda DA, Portes-Junior JA, Neves-Ferreira AG, Santoro ML, Barbaro KC (2013). Cloning, expression and characterization of a phospholipase D from *Loxosceles gaucho* venom gland. Biochimie.

[70] Fernandes-Pedrosa F, Junqueira-de-Azevedo L, Goncalves-de-Andrade RM, Kobashi LS, Almeida DD, Ho PL, Tambourgi DV (2008). Transcriptome analysis of *Loxosceles laeta* (Araneae, Sicariidae) spider venomous gland using expressed sequence tags. BMC Genomics.

[71] Dantas AE, Carmo AO, Horta CC, Leal HG, Oliveira-Mendes BB, Martins AP, Chavez-Olortegui C, Kalapothakis E (2016). Description of Loxtox protein family and identification of a new group of phospholipases D from *Loxosceles similis* venom gland. Toxicon.

[72] Gremski LH, da Silveira RB, Chaim OM, Probst CM, Ferrer VP, Nowatzki J, Weinschutz HC, Madeira HM, Gremski W, Nader HB, et al. A novel expression profile of the Loxosceles intermedia spider venomous gland revealed by transcriptome analysis. Mol Biosyst. 2010;2403–2416. 10.1039/c004118a 20644878

[73] Wille AC, Chaves-Moreira D, Trevisan-Silva D, Magnoni MG, Boia-Ferreira M, Gremski LH, Gremski W, Chaim OM, Senff-Ribeiro A, Veiga SS (2013). Modulation of membrane phospholipids, the cytosolic calcium influx and cell proliferation following treatment of B16-F10 cells with recombinant phospholipase-D from *Loxosceles intermedia* (brown spider) venom. Toxicon.

[74] Machado LF, Laugesen S, Botelho ED, Ricart CA, Fontes W, Barbaro KC, Roepstorff P, Sousa MV (2005). Proteome analysis of brown spider venom: identification of loxnecrogin isoforms in *Loxosceles gaucho* venom. Proteomics.

[75] Kalapothakis E, Chatzaki M, Goncalves-Dornelas H, de Castro CS, Silvestre FG, Laborne FV, de Moura JF, Veiga SS, Chavez-Olortegui C, Granier C, Barbaro KC (2007). The Loxtox protein family in *Loxosceles intermedia* (Mello-Leitao) venom. Toxicon.

[76] Appel MH, da Silveira RB, Chaim OM, Paludo KS, Silva DT, Chaves DM, da Silva PH, Mangili OC, Senff-Ribeiro A, Gremski W, et al. Identification, cloning and functional characterization of a novel dermonecrotic toxin (phospholipase D) from brown spider (*Loxosceles intermedia*) venom. Biochim Biophys Acta*.* 2008; 1780:167–178. 10.1016/j.bbagen.2007.11.007 18082635

[77] Chaim OM, Sade YB, da Silveira RB, Toma L, Kalapothakis E, Chavez-Olortegui C, Mangili OC, Gremski W, von Dietrich CP, Nader HB, Sanches Veiga S (2006). Brown spider dermonecrotic toxin directly induces nephrotoxicity. Toxicol Appl Pharmacol.

[78] da Silveira RB, Pigozzo RB, Chaim OM, Appel MH, Dreyfuss JL, Toma L, Mangili OC, Gremski W, Dietrich CP, Nader HB, Veiga SS (2006). Molecular cloning and functional characterization of two isoforms of dermonecrotic toxin from *Loxosceles intermedia* (brown spider) venom gland. Biochimie.

[79] da Silveira RB, Pigozzo RB, Chaim OM, Appel MH, Silva DT, Dreyfuss JL, Toma L, Dietrich CP, Nader HB, Veiga SS, Gremski W (2007). Two novel dermonecrotic toxins LiRecDT4 and LiRecDT5 from brown spider (*Loxosceles intermedia*) venom: from cloning to functional characterization. Biochimie.

[80] Vuitika L, Gremski LH, Belisario-Ferrari MR, Chaves-Moreira D, Ferrer VP, Senff-Ribeiro A, Chaim OM, Veiga SS (2013). Brown spider phospholipase-D containing a conservative mutation (D233E) in the catalytic site: identification and functional characterization. J Cell Biochem.

[81] Lee S, Lynch KR (2005). Brown recluse spider (*Loxosceles reclusa*) venom phospholipase D (PLD) generates lysophosphatidic acid (LPA). Biochem J.

[82] Ramos-Cerrillo B, Olvera A, Odell GV, Zamudio F, Paniagua-Solis J, Alagon A, Stock RP (2004). Genetic and enzymatic characterization of sphingomyelinase D isoforms from the North American fiddleback spiders *Loxosceles boneti* and *Loxosceles reclusa*. Toxicon.

[83] Lajoie DM, Roberts SA, Zobel-Thropp PA, Delahaye JL, Bandarian V, Binford GJ, Cordes MH (2015). Variable Substrate preference among phospholipase D toxins from Sicariid spiders. J Biol Chem.

[84] Catalan A, Cortes W, Sagua H, Gonzalez J, Araya JE (2011). Two new phospholipase D isoforms of *Loxosceles laeta*: cloning, heterologous expression, functional characterization, and potential biotechnological application. J Biochem Mol Toxicol.

[85] de Santi Ferrara GI, Fernandes-Pedrosa Mde F, Junqueira-de-Azevedo Ide L, Goncalves-de-Andrade RM, Portaro FC, Manzoni-de-Almeida D, Murakami MT, Arni RK, van den Berg CW, Ho PL, Tambourgi DV (2009). SMase II, a new sphingomyelinase D from *Loxosceles laeta* venom gland: molecular cloning, expression, function and structural analysis. Toxicon.

[86] Desai A, Lankford HA, Warren JS (2000). *Loxosceles deserta* spider venom induces the expression of vascular endothelial growth factor (VEGF) in keratinocytes. Inflammation.

[87] Desai A, Miller MJ, Gomez HF, Warren JS (1999). *Loxosceles deserta* spider venom induces NF-kappaB-dependent chemokine production by endothelial cells. J Toxicol Clin Toxicol.

[88] Chatzaki M, Horta CC, Almeida MO, Pereira NB, Mendes TM, Dias-Lopes C, Guimaraes G, Moro L, Chavez-Olortegui C, Horta MC, Kalapothakis E (2012). Cutaneous loxoscelism caused by *Loxosceles similis* venom and neutralization capacity of its specific antivenom. Toxicon.

[89] Silvestre FG, de Castro CS, de Moura JF, Giusta MS, De Maria M, Alvares ES, Lobato FC, Assis RA, Goncalves LA, Gubert IC (2005). Characterization of the venom from the Brazilian brown spider *Loxosceles similis* Moenkhaus, 1898 (Araneae, Sicariidae). Toxicon.

[90] Chaim OM, da Silveira RB, Trevisan-Silva D, Ferrer VP, Sade YB, Boia-Ferreira M, Gremski LH, Gremski W, Senff-Ribeiro A, Takahashi HK (1811). Phospholipase-D activity and inflammatory response induced by brown spider dermonecrotic toxin: endothelial cell membrane phospholipids as targets for toxicity. Biochim Biophys Acta.

[91] Mendes TM, Oliveira D, Figueiredo LF, Machado-de-Avila RA, Duarte CG, Dias-Lopes C, Guimaraes G, Felicori L, Minozzo JC, Chavez-Olortegui C (2013). Generation and characterization of a recombinant chimeric protein (rCpLi) consisting of B-cell epitopes of a dermonecrotic protein from *Loxosceles intermedia* spider venom. Vaccine.

[92] Chaves-Moreira D, Souza FN, Fogaca RT, Mangili OC, Gremski W, Senff-Ribeiro A, Chaim OM, Veiga SS (2011). The relationship between calcium and the metabolism of plasma membrane phospholipids in hemolysis induced by brown spider venom phospholipase-D toxin. J Cell Biochem.

[93] van Meeteren LA, Frederiks F, Giepmans BN, Pedrosa MF, Billington SJ, Jost BH, Tambourgi DV, Moolenaar WH (2004). Spider and bacterial sphingomyelinases D target cellular lysophosphatidic acid receptors by hydrolyzing lysophosphatidylcholine. J Biol Chem.

[94] Chaves-Moreira D, Chaim OM, Sade YB, Paludo KS, Gremski LH, Donatti L, de Moura J, Mangili OC, Gremski W, da Silveira RB (2009). Identification of a direct hemolytic effect dependent on the catalytic activity induced by phospholipase-D (dermonecrotic toxin) from brown spider venom. J Cell Biochem.

[95] Paludo KS, Biscaia SM, Chaim OM, Otuki MF, Naliwaiko K, Dombrowski PA, Franco CR, Veiga SS (2009). Inflammatory events induced by brown spider venom and its recombinant dermonecrotic toxin: a pharmacological investigation. Comp Biochem Physiol C Toxicol Pharmacol.

[96] de Souza AL, Malaque CM, Sztajnbok J, Romano CC, Duarte AJ, Seguro AC (2008). *Loxosceles* venom-induced cytokine activation, hemolysis, and acute kidney injury. Toxicon.

[97] El Alwani M, Wu BX, Obeid LM, Hannun YA (2006). Bioactive sphingolipids in the modulation of the inflammatory response. Pharmacol Ther.

[98] Pettus BJ, Chalfant CE, Hannun YA (2004). Sphingolipids in inflammation: roles and implications. Curr Mol Med.

[99] Horta CC, Oliveira-Mendes BB, Do Carmo AO, Siqueira FF, Barroca TM, dos Santos Nassif Lacerda SM, de Almeida Campos PH, de Franca LR, Ferreira RL, Kalapothakis E (2013). Lysophosphatidic acid mediates the release of cytokines and chemokines by human fibroblasts treated with loxosceles spider venom. J Invest Dermatol.

[100] Lajoie DM, Zobel-Thropp PA, Kumirov VK, Bandarian V, Binford GJ, Cordes MH (2013). Phospholipase D toxins of brown spider venom convert lysophosphatidylcholine and sphingomyelin to cyclic phosphates. PLoS One.

[101] Zobel-Thropp PA, Kerins AE, Binford GJ (2012). Sphingomyelinase D in sicariid spider venom is a potent insecticidal toxin. Toxicon.

[102] da Silveira RB, Wille AC, Chaim OM, Appel MH, Silva DT, Franco CR, Toma L, Mangili OC, Gremski W, Dietrich CP (2007). Identification, cloning, expression and functional characterization of an astacin-like metalloprotease toxin from *Loxosceles intermedia* (brown spider) venom. Biochem J.

[103] Trevisan-Silva D, Gremski LH, Chaim OM, da Silveira RB, Meissner GO, Mangili OC, Barbaro KC, Gremski W, Veiga SS, Senff-Ribeiro A (2010). Astacin-like metalloproteases are a gene family of toxins present in the venom of different species of the brown spider (genus *Loxosceles*). Biochimie.

[104] Trevisan-Silva D, Bednaski AV, Gremski LH, Chaim OM, Veiga SS, Senff-Ribeiro A (2013). Differential metalloprotease content and activity of three *Loxosceles* spider venoms revealed using two-dimensional electrophoresis approaches. Toxicon.

[105] Matsubara FH, Gremski LH, Meissner GO, Constantino Lopes ES, Gremski W, Senff-Ribeiro A, Chaim OM, Veiga SS (2013). A novel ICK peptide from the *Loxosceles intermedia* (brown spider) venom gland: cloning, heterologous expression and immunological cross-reactivity approaches. Toxicon.

[106] Meissner GO, de Resende Lara PT, Scott LP, Braz AS, Chaves-Moreira D, Matsubara FH, Soares EM, Trevisan-Silva D, Gremski LH, Veiga SS, Chaim OM (2016). Molecular cloning and in silico characterization of knottin peptide, U2-SCRTX-Lit2, from brown spider (*Loxosceles intermedia*) venom glands. J Mol Model.

[107] Zela SP, Fernandes Pedrosa MF, Murakami MT, De Andrade SA, Arni RK, Tambourgi DV (2004). Crystallization and preliminary crystallographic analysis of SMase I, a sphingomyelinase from *Loxosceles laeta* spider venom. Acta Crystallogr D Biol Crystallogr.

[108] de Giuseppe PO, Ullah A, Silva DT, Gremski LH, Wille AC, Chaves Moreira D, Ribeiro AS, Chaim OM, Murakami MT, Veiga SS, Arni RK (2011). Structure of a novel class II phospholipase D: catalytic cleft is modified by a disulphide bridge. Biochem Biophys Res Commun.

[109] Murakami MT, Fernandes-Pedrosa MF, de Andrade SA, Gabdoulkhakov A, Betzel C, Tambourgi DV, Arni RK (2006). Structural insights into the catalytic mechanism of sphingomyelinases D and evolutionary relationship to glycerophosphodiester phosphodiesterases. Biochem Biophys Res Commun.

[110] Murakami MT, Fernandes-Pedrosa MF, Tambourgi DV, Arni RK (2005). Structural basis for metal ion coordination and the catalytic mechanism of sphingomyelinases D. J Biol Chem.

[111] Ullah A, Magalhaes GS, Masood R, Mariutti RB, Coronado MA, Murakami MT, Barbaro KC, Arni RK (2014). Crystallization and preliminary X-ray diffraction analysis of a novel sphingomyelinase D from *Loxosceles gaucho* venom. Acta Crystallogr F Struct Biol Commun.

[112] Ullah A, de Giuseppe PO, Murakami MT, Trevisan-Silva D, Wille AC, Chaves-Moreira D, Gremski LH, da Silveira RB, Sennf-Ribeiro A, Chaim OM (2011). Crystallization and preliminary X-ray diffraction analysis of a class II phospholipase D from L*oxosceles intermedia* venom. Acta Crystallogr Sect F: Struct Biol Cryst Commun.

[113] Coronado MA, Ullah A, da Silva LS, Chaves-Moreira D, Vuitika L, Chaim OM, Veiga SS, Chahine J, Murakami MT, Arni RK. Structural insights into substrate binding of brown spider venom class II phospholipases D. Curr Protein Pept Sci. 2015. 10.2174/1389203716666150505231625 25961401

[114] Vuitika L, Chaves-Moreira D, Caruso I, Lima MA, Matsubara FH, Murakami MT, Takahashi HK, Toledo MS, Coronado MA, Nader HB (1861). Active site mapping of *Loxosceles* phospholipases D: Biochemical and biological features. Biochim Biophys Acta.

[115] Catalan A, Cortes W, Munoz C, Araya JE (2014). Tryptophan and aspartic acid residues present in the glycerophosphoryl diester phosphodiesterase (GDPD) domain of the *Loxosceles laeta* phospholipase D are essential for substrate recognition. Toxicon.

[116] Flores-Diaz M, Monturiol-Gross L, Naylor C, Alape-Giron A, Flieger A (2016). Bacterial sphingomyelinases and phospholipases as virulence factors. Microbiol Mol Biol Rev.

[117] da Silva PH, Hashimoto Y, dos Santos FA, Mangili OC, Gremski W, Veiga SS (2003). Hematological cell findings in bone marrow and peripheral blood of rabbits after experimental acute exposure to *Loxosceles intermedia* (brown spider) venom. Toxicon.

[118] Kusma J, Chaim OM, Wille AC, Ferrer VP, Sade YB, Donatti L, Gremski W, Mangili OC, Veiga SS (2008). Nephrotoxicity caused by brown spider venom phospholipase-D (dermonecrotic toxin) depends on catalytic activity. Biochimie.

[119] Paludo KS, Gremski LH, Veiga SS, Chaim OM, Gremski W, de Freitas BD, Nader HB, Dietrich CP, Franco CR (2006). The effect of brown spider venom on endothelial cell morphology and adhesive structures. Toxicon.

[120] Ribeiro RO, Chaim OM, da Silveira RB, Gremski LH, Sade YB, Paludo KS, Senff-Ribeiro A, de Moura J, Chavez-Olortegui C, Gremski W (2007). Biological and structural comparison of recombinant phospholipase D toxins from *Loxosceles intermedia* (brown spider) venom. Toxicon.

[121] Chaves-Moreira D, de Moraes FR, Caruso IP, Chaim OM, Senff-Ribeiro A, Ullah A, da Silva LS, Chahine J, Arni RK, Veiga SS. Potential implications for designing drugs against the brown spider venom phospholipase-D. J Cell Biochem. 2016. 10.1002/jcb.25678 27563734

[122] Ferrer VP, de Mari TL, Gremski LH, Trevisan Silva D, da Silveira RB, Gremski W, Chaim OM, Senff-Ribeiro A, Nader HB, Veiga SS (2013). A novel hyaluronidase from brown spider (*Loxosceles intermedia*) venom (Dietrich's Hyaluronidase): from cloning to functional characterization. PLoS Negl Trop Dis.

[123] Sade YB, Boia-Ferreira M, Gremski LH, da Silveira RB, Gremski W, Senff-Ribeiro A, Chaim OM, Veiga SS (2012). Molecular cloning, heterologous expression and functional characterization of a novel translationally-controlled tumor protein (TCTP) family member from *Loxosceles intermedia* (brown spider) venom. Int J Biochem Cell Biol.

[124] da Silveira RB, Chaim OM, Mangili OC, Gremski W, Dietrich CP, Nader HB, Veiga SS (2007). Hyaluronidases in *Loxosceles intermedia* (Brown spider) venom are endo-beta-N-acetyl-d-hexosaminidases hydrolases. Toxicon.

[125] dos Santos LD, Dias NB, Roberto J, Pinto AS, Palma MS (2009). Brown recluse spider venom: proteomic analysis and proposal of a putative mechanism of action. Protein Pept Lett.

[126] Lane L, McCoppin HH, Dyer J (2011). Acute generalized exanthematous pustulosis and Coombs-positive hemolytic anemia in a child following *Loxosceles reclusa* envenomation. Pediatr Dermatol.

[127] Makris M, Spanoudaki N, Giannoula F, Chliva C, Antoniadou A, Kalogeromitros D (2009). Acute generalized exanthematous pustulosis (AGEP) triggered by a spider bite. Allergol Int.

